# Simultaneous transcatheter aortic and mitral valve-in-valve replacement: A case report

**DOI:** 10.1097/MD.0000000000045726

**Published:** 2026-01-16

**Authors:** Yue Mao, Yang Zhan, Rong Li, Jingjing Chen, Quanfeng Mo

**Affiliations:** a Department of Nursing, The Second Affiliated Hospital of Zhejiang University School of Medicine, Hangzhou, China.

**Keywords:** case report, transcatheter aortic valve replacement, transcatheter mitral valve replacement, valve-in-valve

## Abstract

**Rationale:**

There are fewer reports in China about re-performing combined transcatheter transcatheter aortic and mitral valve-in-valve replacement after bioprosthetic valve failure. Due to the higher difficulty of the combined secondary bivalve procedure and the greater risk of pericardial tamponade and malignant arrhythmias, the professional demands on the surgeon and team are extremely high. It is particularly important to provide quality perioperative care for such patients.

**Patient concerns:**

This study reports a case of bioprosthetic valve failure after undergoing transcatheter aortic and mitral bicuspid valve-in-valve replacement.After active treatment and careful care, the patient recovered well after the operation and had a good quality of life during the 1 year follow-up period.

**Diagnoses:**

Valve function was measured by 3-dimensional echocardiography.

**Interventions:**

The key points of perioperative care include: close monitoring of the patient’s condition and provision of first aid before surgery; close monitoring of hemodynamic parameters, management of fluid balance, provision of respiratory support and oxygen therapy management, prevention of complications and early cardiac rehabilitation after surgery.

**Outcomes:**

On the 16th day after the operation, the 3-dimensional echocardiogram showed that the mitral regurgitation was slight. Mitral regurgitation was slight, and aortic valve flow velocity and differential pressure were normalized. At 1 year follow-up, there were no adverse cardiovascular events and quality of life improved.

**Lessons:**

For patients undergoing simultaneous transcatheter bivalve replacement with multiple comorbidities, focus should be placed on constructing an integrated perioperative intervention pathway to comprehensively improve clinical tolerance and control the risk of complex cases.

## 1. Introduction

In China, surgical valve replacement remains the preferred modality for the treatment of heart valve disease. Due to the advantage of not requiring lifelong anticoagulation therapy,^[1]^ more and more patients are opting for bioprosthetic valves. However, the limited durability of these valves, which typically deteriorate within 10 to 15 years, often requires reoperation.^[2]^ At this point, if reopening surgery is required, not only do they face high technical difficulties and risks, but the perioperative mortality rate is more than 20%.^[3]^ In addition, about half of the patients with structural valve degeneration are considered unable to undergo repeat surgery due to comorbidities or frailty.^[4,5]^ Transcatheter valve-in-valve (ViV) replacement has emerged as a minimally invasive and highly effective alternative to reoperation by implanting a novel bioprosthetic valve inside the diseased valve. Its advantages include reduced surgical trauma and faster recovery.^[4,5]^ Transcatheter aortic valve-in-valve replacement (ViV-TAVR) and transcatheter mitral valve-in-valve replacement (ViV-TMVR) are increasingly being used as novel strategies for the treatment of degenerative aortic and mitral bioprostheses, respectively.^[6,7]^ However, the procedure still carries the risk of complications such as hematoma at the vascular puncture site, valve positioning excursion, guidewire injury leading to left ventricular rupture, and medically induced atrial septal defects.^[1]^ The synchronized transcatheter mitral and aortic valve simultaneous replacement reported in this paper is relatively rare. Simultaneous minimally invasive intervention of the aortic and mitral valves has unique pathophysiologic interactions (interdependent loading conditions, dynamic changes in atrial pressures, risk of left ventricular outflow tract obstruction, and thromboembolic potential) that make it more difficult to treat and care for compared with single valve surgery. This study was approved by the Ethics Committee and Institutional Review Board of the Second Affiliated Hospital of Zhejiang University School of Medicine, and obtain informed consent from the patient.

## 2. Case report

A 65-year-old Chinese woman presented to our hospital with “chest tightness, dyspnea, and bilateral lower extremity edema lasting 2 months after bioprosthetic valve replacement.” 13 years ago, the patient underwent bioprosthetic valve replacement (mitral and aortic) and tricuspid valve repair at another hospital, and had a good recovery after the operation. Admission echocardiogram showed severe mitral stenosis with severe regurgitation, moderate aortic stenosis, moderate tricuspid regurgitation, severe pulmonary hypertension, total heart enlargement and left atrial fibrillation thrombus. Left ventricular ejection fraction was 40.9%. Electrocardiogram showed atrial fibrillation. Laboratory findings: troponin T 0.048 ng/mL, B-type natriuretic peptide 169.1 pg/mL, international normalized ratio (INR) 3.35, and serum creatinine 214.3 μmol/L. Past medical history included 20 years of atrial fibrillation, 5 years of hyperthyroidism, and 6 months of diabetes mellitus; long-term oral medications of methimazole, levothyroxine sodium, and gliclazide; penicillin History of allergy. During hospitalization, the patient suffered from recurrent acute heart failure, which was mainly manifested by severe dyspnea and obvious depressed edema of both lower limbs.

## 3. Treatment and care

Given the patient’s critical condition and the extremely high risk of secondary open-heart surgery, the valve team finally decided to urgently implement the combined VIV-TAVR + VIV-TMVR treatment program after a multidisciplinary consultation. The patient was operated under general anesthesia. The patient was given a right internal jugular vein puncture and a temporary pacemaker was placed. The left and right femoral arteries and femoral veins were punctured under ultrasound guidance. 6F and 7F sheaths were placed in the right femoral artery and left femoral artery, respectively; a 7F sheath was placed in the right femoral vein, and a 6F sheath was placed in the left femoral vein, followed by pre-interposition of a ProGlide vascular stapler in the left femoral artery, and a ProGlide in the right femoral vein. transesophageal ultrasound showed aortic stenosis, and severe mitral stenosis with severe closure insufficiency. A 6F pigtail catheter was placed in the left femoral artery to the aortic root, and a 6F AL1.0 contrast catheter was introduced into the right femoral artery to the aortic arch, exchanged for a super stiff guidewire, and a 14F sheath was placed. Coronary angiography suggested that the right coronary artery did not have significant stenosis, and a straight-ended transvalvular guidewire was placed to the left ventricle, and exchanged for a super stiff guidewire. A left ventricular and aortic pressure step difference of 35 mm Hg was measured, and an ultra-stiff guidewire was exchanged for a super stiff guidewire via a pigtail catheter to the left ventricle. The Sapein3 23 mm valve was placed under rapid pacing at 200 beats/min. Repeated imaging was performed to localize the Sapein3 23-mm valve in a satisfactory position, and the valve was successfully released and not recovered, and the coronary artery was patent on repeat imaging. Posterior ultrasound-assisted successful puncture of the atrial septum. A pigtail catheter was guided across the mitral valve, and an ultrarigid guidewire was introduced to the left ventricle. A large 16F sheath was introduced through the ultrarigid guidewire, and a Sapien3 29-mm valve was inserted. Gradual release was performed and the valve was successfully released. The valve was dilated once afterward. After the disappearance of mitral regurgitation and stenosis on ultrasound, the large sheath was withdrawn, and cerebral arteriography was performed without significant cerebral artery embolism, and the left and right femoral arteries and the right femoral vein were occluded by ProGlide, with no bleeding from the puncture wound. No significant stenosis was seen on right femoral arteriography. Postoperatively, he was sent to the intensive care unit, where mechanical ventilation via endotracheal intubation was initiated and blood pressure stabilization was maintained with norepinephrine. The comprehensive treatment plan included infection control with imipenem-cestatin sodium, anticoagulation with low molecular heparin, and promotion of diuresis with bumetanide combined with furosemide.

The patient was switched to mask oxygen administration (5 L/min) after removal of tracheal intubation on the third postoperative day, but suddenly developed chest tightness and dyspnea 3 hours later, and again showed signs of acute heart failure. Morphine, methylprednisolone sodium succinate and levosimendan were administered, and high-flow oxygen therapy (45 L/min, Fraction of inspiration O_2_ 40%) was initiated simultaneously. Percutaneous arterial oxygen saturation (SpO2), respiratory rate and heart rate were continuously monitored, and oxygen therapy parameters were dynamically adjusted to maintain SpO_2_ > 92%, and then changed to nasal cannula oxygenation (5 L/min) on the ninth postoperative day. The nurse in charge checked the function of the oxygen therapy device daily and maintained the airway patency to prevent oxygenation abnormality due to equipment malfunction.

A graded nurse-led monitoring program was implemented in the postoperative period: continuous electrocardiographic monitoring combined with invasive arterial blood pressure monitoring; urine output was recorded hourly, and the physician was notified immediately if it fell below 0.5 mL/kg/h for 2 consecutive hours. The preset SpO_2_ alarm threshold was 92%, and bedside assessment was required within 5 minutes after triggering. Vasoactive drug use was standardized to MAP < 65 mm Hg or systolic blood pressure <90 mm Hg, and patients received norepinephrine (2 mg, 2 mL/h) with epinephrine (1 mg, 5 mL/h) to maintain systolic blood pressure of 100 to 130 mm Hg, which was discontinued until postoperative day 3. Fluid management was strictly controlled with an infusion rate of 15 to 45 drops/minute, ensuring a negative 24-hour balance of 0 to 500 mL through a standardized access chart.Laboratory monitoring consisted of daily BNP/electrolyte testing for 72 hours postoperatively and serum creatinine tracking for the first 5 days.

Postoperative complications require vigilance: prevention of pericardial tamponade: postoperative hemodynamic monitoring is continuous, and in the event of chest pain, dyspnea, or a sudden drop in blood pressure of > 30%, an emergency procedure is initiated and a sterile pericardiocentesis kit is prepared for use. Arrhythmia prevention: A temporary pacemaker (60 bpm, 10 mA output) was implanted intraoperatively, and electrocardiograms were performed in the immediate postoperative period, 4 hours and 24 hours after surgery, and electrolyte levels were tracked daily. On the fourth postoperative day, the blood potassium dropped to 2.8 mmol/L, 10% potassium chloride was given orally and potassium-preserving diuretics were changed, and the blood potassium was normalized at the time of discharge. The safe management of anticoagulation was based on a systematic strategy: daily monitoring of INR, platelets and D-dimer; establishment of an immediate reporting mechanism for bleeding symptoms; and individualized adjustment of anticoagulation intensity based on renal function and baseline INR. The patient’s INR was maintained at 1.19 to 1.44, and with active prevention, no relevant postoperative complications occurred in this case.

## 4. Discussion

VIV technique has been shown to be effective in the treatment of structural failure of bioprosthetic valves; however, the number of simultaneous VIV-TAVR and VIV-TMVR performed worldwide is relatively low,^[8]^ and is extremely rare in China. This is due not only to the strict anatomical and clinical indications that patients need to fulfill but also to the high demands placed on the skills of the operator and the experience of the team.

Biologic valve failure involves multiple mechanisms such as structural deterioration (calcification, tearing), infection, or thrombosis, and patients with bicuspid valve failure are often comorbid with underlying diseases such as heart failure and pulmonary hypertension, resulting in extreme hemodynamic instability.^[8]^ The simultaneous management of aortic and mitral valve degeneration requires precise assessment of the nature of the lesions, the degree of degeneration, and the interplay between the 2 valves. In this case, the patient required valve-in-valve replacement due to bioprosthetic valve failure, which required precise traversal of the original valve during surgical maneuvers while avoiding damage to the conduction system and other cardiac structures. The size matching and positioning requirements are extremely high to avoid serious complications such as perivalvular leakage, left ventricular outflow tract obstruction, or new valve detachment.^[9]^ In addition, the risk of perioperative pericardial tamponade and malignant arrhythmias is significantly higher, such as acute heart failure that may still occur after TMVR, requiring vigilance and prompt management.^[10]^ In order to minimize the risk of pericardial tamponade, our medical team developed a comprehensive in-hospital emergency response plan after several preliminary workshops and in conjunction with expert consensus^[11]^ and other professional guidelines. The program ensures tacit teamwork and clear delineation of responsibilities in times of crisis by requiring the medical team to conduct mandatory preoperative multidisciplinary simulation exercises.

TAVR carries the risk of conduction disturbances due to compression of the Hitchcock bundle by the prosthetic annulus. However, studies have shown that the incidence of new conduction block after repeat TAVR is comparable to that of patients with native valve TAVR (10%), suggesting that standard post-TAVR conduction monitoring protocols are applicable.The incidence of permanent pacemaker implantation after VIV-TAVR is approximately 4.2%,^[12]^ and patients undergoing VIV-TMVR are more likely to develop atrial fibrillation, premature ventricular beats and ventricular tachycardia, among other arrhythmias,^[13]^ and these symptoms may lead to dizziness, fatigue, chest tightness, hypotension, and even sudden cardiac death. Also, septal puncture increases the risk of conduction block and arrhythmias. In this case, a temporary pacemaker was implanted intraoperatively. An electrocardiogram was performed immediately after the operation and monitored again at 4 and 24 hours postoperatively. Electrolyte levels including serum potassium, magnesium and calcium were closely monitored to prevent electrolyte-induced arrhythmias.

To improve surgical outcomes, a 3-phase prehabilitation was implemented preoperatively,^[14]^ including cognitive-behavioral interventions^[15]^ combined with structured education,^[16]^ individualized low-intensity aerobic combined with inspiratory muscle training, and daily protein intake of ≥ 1.2 g/kg. postoperative rehabilitation was based on a stepwise protocol^[17]^: passive ankle pumping exercises (10-second plantarflexion/ dorsiflexion + rotation, 15–20 repetitions/group, 3–4 groups/day) were initiated at 6 hours postoperatively. On postoperative day 5, hemodynamic stabilization was achieved and bedside standing and walking exercises were initiated. The final Hospital Anxiety and Depression Scale score decreased from 13 to 8; the activities of daily living score rose to 60 at discharge, which was higher than the admission baseline (45).

In addition, the successful implementation of this case validates the irreplaceable nature of the multidisciplinary cardiac teamwork model. During the procedure, the anesthesia team precisely regulated the hemodynamic balance under the critical state of dual involvement of the body circulation and pulmonary circulation; the intervention team realized the precise positioning and release of the valve device through real-time ultrasound guidance and electrophysiological regulation techniques. The cardiac ultrasound team is responsible for preoperative assessment, intraoperative real-time image guidance, and postoperative valve function assessment; meanwhile, the nursing team also plays an important role in executing medical orders, coordinating communication, establishing a continuous physiological homeostasis monitoring system, patient preparation safety and security, and recognizing emergencies.

Of note, our previous echocardiographic assessment of patients focused on static indices, such as left ventricular ejection fraction and valve transvalvular pressure difference, which may underestimate true cardiac dynamic reserve under stressful conditions. It has been shown that in patients with mild to moderate aortic stenosis, an exercise-induced increase in E/e′ ≥15 is significantly associated with adverse cardiac outcomes.^[18]^ This finding highlights the prognostic importance of dynamic diastolic parameters under stressful conditions. Similarly, recent evidence suggests that resting cardiac power output and its exercise-induced alterations are independent predictors of death and hospitalization for heart failure after TAVI.^[19]^ Thus, the integration of such dynamic echocardiographic measures into perioperative assessments may provide a more comprehensive understanding of cardiac functional reserve, leading to individualized monitoring and management strategies for patients undergoing complex transcatheter valve-in-valve procedures.

## 5. Conclusion

We report a case of simultaneous VIV-TAVR and VIV-TMVR after bioprosthetic valve failure. Our report enriches the database of this rare clinical case and provides relevant experience in treatment and care. At 1 year follow-up, there were no adverse cardiovascular events and quality of life improved. From the optimization of preoperative psycho-physiological reserve, precise surgical operation, to the refined management of circulatory-respiratory function in the postoperative period, and up to the advancement of a stepped functional rehabilitation program, together they constitute the core framework for enhancing clinical tolerance, avoiding the risk of complications, and accelerating functional reconstruction. In future studies, more attention should be paid to this rare case.

## Acknowledgments

We would like to thank all the members who participated in this study.

## Author contributions

**Methodology:** Yang Zhan.

**Supervision:** Jingjing Chen, Quanfeng Mo.

**Validation:** Jingjing Chen, Quanfeng Mo.

**Writing – original draft:** Yue Mao.

**Writing – review & editing:** Yue Mao, Yang Zhan, Rong Li.
